# Breed Ancestry, Divergence, Admixture, and Selection Patterns of the Simbra Crossbreed

**DOI:** 10.3389/fgene.2020.608650

**Published:** 2021-01-28

**Authors:** Magriet A. van der Nest, Nompilo Hlongwane, Khanyisile Hadebe, Wai-Yin Chan, Nicolaas A. van der Merwe, Lieschen De Vos, Ben Greyling, Bhaveni B. Kooverjee, Pranisha Soma, Edgar F. Dzomba, Michael Bradfield, Farai C. Muchadeyi

**Affiliations:** ^1^Biotechnology Platform, Agricultural Research Council, Pretoria, South Africa; ^2^Department of Biochemistry, Genetics and Microbiology, University of Pretoria, Pretoria, South Africa; ^3^Animal Production, Agricultural Research Council, Pretoria, South Africa; ^4^Discipline of Genetics, School of Life Sciences, University of KwaZulu-Natal, Durban, South Africa; ^5^Agricultural Business SA, Pretoria, South Africa

**Keywords:** simbra, crossbreeding, genomic selection, indicine, taurine

## Abstract

In this study, we evaluated an admixed South African Simbra crossbred population, as well as the Brahman (Indicine) and Simmental (Taurine) ancestor populations to understand their genetic architecture and detect genomic regions showing signatures of selection. Animals were genotyped using the Illumina BovineLD v2 BeadChip (7K). Genomic structure analysis confirmed that the South African Simbra cattle have an admixed genome, composed of 5/8 Taurine and 3/8 Indicine, ensuring that the Simbra genome maintains favorable traits from both breeds. Genomic regions that have been targeted by selection were detected using the linkage disequilibrium-based methods iHS and Rsb. These analyses identified 10 candidate regions that are potentially under strong positive selection, containing genes implicated in cattle health and production (e.g., TRIM63, KCNA10, NCAM1, SMIM5, MIER3, and SLC24A4). These adaptive alleles likely contribute to the biological and cellular functions determining phenotype in the Simbra hybrid cattle breed. Our data suggested that these alleles were introgressed from the breed's original indicine and taurine ancestors. The Simbra breed thus possesses derived parental alleles that combine the superior traits of the founder Brahman and Simmental breeds. These regions and genes might represent good targets for *ad-hoc* physiological studies, selection of breeding material and eventually even gene editing, for improved traits in modern cattle breeds. This study represents an important step toward developing and improving strategies for selection and population breeding to ultimately contribute meaningfully to the beef production industry.

## Introduction

Cattle play an important part in the agricultural economy worldwide. Modern cattle were derived from at least two independent domestication events that gave rise to two subspecies of cattle (Loftus et al., 1994; Ajmone-Marsan et al., 2010). The one is humpless Taurine (*Bos taurus taurus*) cattle, with *Bos primigenius primigenius* ancestry, which was domesticated ~10,500 years ago in Eastern Europe. The other is the humped zebu or Indicine (*Bos taurus indicus*) cattle, with *Bos primigenius namadicus* ancestry, which was domesticated ~7,000 years ago in India (Bradley et al., 1996). Domestication of cattle resulted in animals with high overall genetic and phenotypic variability (Taberlet et al., 2008).

The rise of the “breed” concept, and associated intensive artificial selection, had resulted in specialized cattle breeds that underwent further organized selection to enhance production and adaptability (Iso-Touru et al., 2016). Taurine breeds have been intensively selected for milk and meat yield (Low et al., 2020). For example, selection for traits associated with meat production (e.g., fast growth, carcass quality, meat quality, and meat yield) and increased fertility gave rise to Simmental, which is the oldest and one of the most widespread Taurine beef breeds (Bordbar et al., 2020; Ríos-Utrera et al., 2020). In contrast, selection for high tolerance to parasites, heat resistance and overall hardiness gave rise to Indicine breeds, such as Brahman, the first beef cattle breed developed in the United States (Dikmen et al., 2018).

Various crossbreeds have also been developed to improve environmental adaptability and desirable performance (Paim et al., 2020). These cattle breeds combine the favorable traits/genes that characterized their purebred parental breeds. An added benefit inherent of crossbreeding is heterosis or hybrid vigor that may give rise to qualities that are more superior in the crossbreed than its parental inbred lines (Harrison and Larson, 2014; Frankham, 2015; Gouws, 2017). Furthermore, crossbreeding remains an important mechanism for increasing the overall genetic variation of modern cattle breeds (Kristensen et al., 2015), especially given the substantial losses incurred due to intensive selection for improved productivity and adaptability (Albertí et al., 2008; Taberlet et al., 2008). However, despite these benefits, it is still unclear whether the genetic composition of a crossbreed is stable over time (Paim et al., 2020). It is also not known if crossbreeding may cause reduction in performance and fitness due to genetic erosion and outbreeding depression (Harrison and Larson, 2014; Frankham, 2015; Gouws, 2017). Genetic erosion may cause reduction in performance since genetic diversity is necessary for evolution to occur, while loss of genetic diversity is related to inbreeding that reduces reproductive fitness (Reed and Frankham, 2003).

The Simbra crossbreed was developed in the United States in the late 1960s, shortly after the first Simmental arrived from Europe (Gouws, 2016). It has been described as the “all-purpose American breed “and was developed by hybridization of the Brahman and Simmental breeds (Gouws, 2016). Generally, crossbreeding of Brahman with Taurine breeds produces hardy animals with better meat quality than purebred Brahmans (Crouse et al., 1989; Johnson et al., 1990; Schatz et al., 2014). The high tolerance of Simbra to harsh conditions (e.g., heat, humidity, parasites, seasonally poor pasture quality, and large distances required to be walked while grazing) is thus derived from its Brahman parentage. In turn, its good meat quality (e.g., carcass composition and conformation), early sexual maturity, milking ability, rapid growth, and docile temperament are attributed to its Simmental ancestry (Smith, 2010). Although Simbra cattle are mainly produced in the USA, the breed was also introduced to other countries. For example, Simbra was introduced to South Africa in the late 1990s where it is among the 10 most popular breeds in the country (Scholtz et al., 2008). Several population studies provided insight regarding genetic structure of popular South African cattle breeds (e.g., Simmental, Afrikaner and Nguni) (Bennett and Gregory, 1996; Pico, 2004; Martínez and Galíndez, 2006; Greyling et al., 2008; Sanarana et al., 2016; Pienaar et al., 2018). However, little is known about the genetic diversity and population structure within and between South African Simbra and the ancestral Brahman and Simmental breeds.

Various studies showed that information mined from whole genome data is useful for estimating proportional ancestry, maximizing genetic variability and for developing breeding strategies (Kim et al., 2017; Sharma et al., 2017; Bhati et al., 2020). In other words, knowledge emerging from genomic studies can be used to improve livestock in terms of meat and milk production, disease resistance and reproductive health (Kim et al., 2017; Sharma et al., 2017; Bhati et al., 2020). For example, genome-wide association studies (GWAS) have been used to identify genes involved in meat quality in different Taurine (Gutiérrez-Gil et al., 2008; McClure et al., 2012; Allais et al., 2014; Xia et al., 2016), Indicine (Tizioto et al., 2013; Magalhães et al., 2016), and crossbreeds (Bolormaa et al., 2011; Lu et al., 2013; Hulsman et al., 2014). Genome-based selection strategies are thus increasingly regarded as invaluable for ultimately improving cattle fitness, productivity, and quality (Daetwyler et al., 2014; Kim et al., 2017).

The overall goal of this study was to estimate the adaptive potential of the Indicine- and Taurine-derived genomic components in the South African Simbra cattle breed. We therefore aimed to (i) determine levels of heterozygosity; (ii) infer the overall population structure and admixture ancestry in Simbra cattle; (iii) and identify genomic regions subject to positive selection and to associate these with putative productivity and adaptive traits. For this purpose, Simbra, Brahman and Simmental animals were genotyped using the cost-effective Illumina's low density Bovine BeadArray (7K) technology that allows the genotyping of a larger number of individuals, as part of the South African Beef Genomics Project. Several studies have successfully used this approach in genome-wide association studies as genotyping large numbers of individuals with thousands of SNPs remains prohibitively expensive for many research groups. The data generated in this study will be instrumental for informing and designing appropriate management and breeding strategies for maximizing Simbra productivity in South Africa and cattle in general.

## Materials and Methods

### Animals

**Figure 1:**
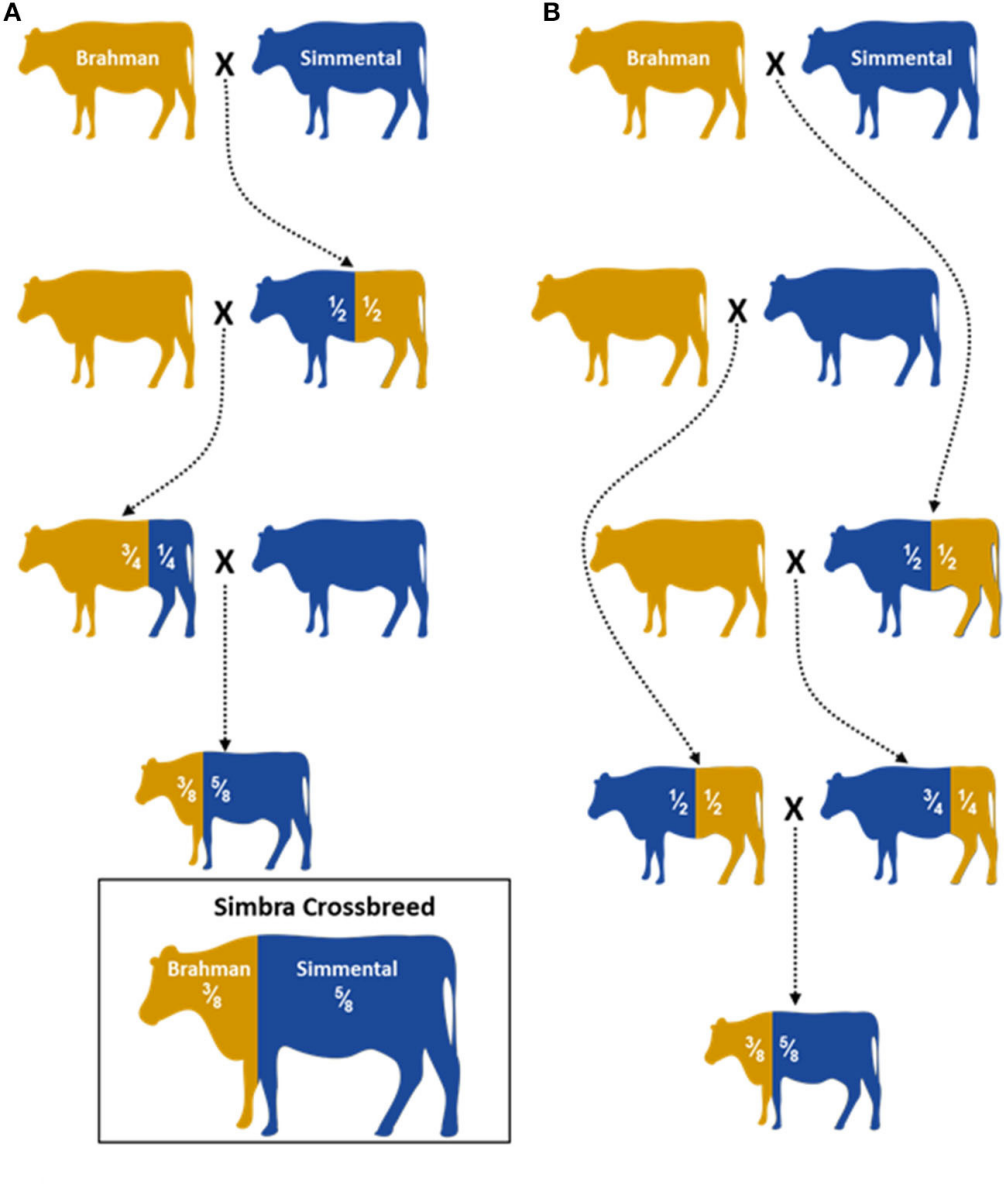
Illustration of two hybridization schemes **(A)** and **(B)** used to establish the Simbra crossbreed (adopted from Paim et al., 2020). A 5/8 Simmental and 3/8 Brahman are the optimum composition needed to retain the favorable traits both parental breeds (O' Connor et al., 1997; Smith, 2010). Controlled breeding programs are used to establish the next Simbra generations with the optimum composition.

A total of 321 animals were genotyped in this study. These included animals from the South African Simbra crossbred population (Simbra, *n* = 69), as well as Brahman (*Bos taurus indicus, n* = 161) and Simmental (*Bos taurus taurus*, Simmental *n* = 91) populations. These animals were part of stud breeding programs aimed at producing registered Simbra (3/8 Brahman, 5/8 Simmental; Figure 1) that is registered in a herdbook, Brahman and Simmental cattle and were not part of a designed experiment. They were selected based on phenotypic appearance, which was consistent with typical breed characteristics and pedigree information accepted by local breeders and breed societies.

### SNP Genotyping and Quality Control

Genomic DNA was extracted at the ARC-Biotechnology Platform from blood/hair root samples using Qiagen's DNeasy extraction kit (Qiagen, Valencia, CA). The quality and quantity of the DNA were estimated using a Qubit® 2.0 fluorometer (Life Technologies, ThermoFisher Scientific, USA), Nanodrop 1000 spectrophotometer (Nanodrop Technologies, Wilmington, DE), and agarose gel electrophoresis. These DNAs were then used in genotyping experiments at the ARC-Biotechnology Platform as part of the SA Beef Genomics Project during the period 2015–2018. This was done using the Illumina BovineLD v2 BeadChip (7K) (Illumina, San Diego, CA), which features 7,931 single nucleotide polymorphism (SNP) probes that are distributed across the whole bovine genome, with <3 kilobase pair (kb) median gap spacing. Samples were processed according to the Illumina Infinium-II assay protocol (Illumina, Inc. San Diego, CA, 92122, USA). Only autosomal chromosomes were used, and SNP quality control was assessed using PLINK (Purcell et al., 2007). SNPs with a call rate <95% and minor allele frequencies (MAF) <5% across all breeds were removed. SNPs with a high linkage disequilibrium (LD) at a threshold of LD ≥0.8 were also pruned. The SNP & Variation Suite v.8.8.3 (Golden Helix Inc., Bozeman, MT, USA; www.goldenhelix.com) was used to estimate the identity-by-descent (IBD) values between pairs of individuals that can be used to detect and remove related and duplicate samples.

### Genetic Diversity

Various analytical tools were used to estimate the genetic diversity among the Simbra, Brahman and Simmental populations. The observed heterozygosity estimates for each population, as an indication of within-breed diversity, were calculated from observed genotype frequencies obtained from PLINK (Purcell et al., 2007). Here, observed heterozygosity was calculated as (N - O)/N, where N is the number of “non-missing genotypes” for a given individual and O is the number of observed homozygous genotypes for that individual. We also estimated the inbreeding coefficient (F) as a measure of “excess” homozygosity using the SNP & Variation Suite.

### Population Structure

Principal Components Analysis (PCA) (Patterson et al., 2006) and fastSTRUCTURE (Raj et al., 2014) analyses were used to identify patterns of admixture and relatedness among the Simbra cattle, in relation to the Simmental and Brahman populations. PCA was performed using the EIGENSTRAT methodology embedded in the SNP & Variation Suite. The fastSTRUCTURE analysis employed an admixture model and two clusters (*K* = 2; based on the number of ancestral populations) (Smith, 2010). The analysis was executed using independent allele frequencies, and a burn-in of 100 000 iterations, followed by 1 000 000 Markov Chain Monte Carlo iterations. Graphical display of the admixture output was generated using Distruct v1.1 (http://web.stanford.edu/group/rosenberglab/distruct.html).

Local ancestry for admixed Simbra animals were inferred using PCAdmix (Brisbin et al., 2012), which uses PCA to determine the posterior probabilities for the ancestry of a genomic region along each chromosome. More specifically, PCAdmix classifies blocks of SNPs by ancestry through PCA, projecting the loadings of admixed individuals based on the loadings of putative ancestors. It employs a Hidden Markov Model (HMM) to smooth the results and returns the posterior probabilities of ancestry affiliation for each block from the HMM (Brisbin et al., 2012).

To prepare input files for PCAdmix, haplotypes were built using Beagle 5.1 by phasing and imputing missing genotypes from the SNP unphased data (Browning et al., 2018). Chromosomes for each individual in a population were artificially strung together to create two haploid genomes for the individual to increase the amount of information used for PCA. Since PCAdmix requires predefined ancestral groups, we selected two main ancestral groups (Simmental and Brahman cattle) for the Simbra cattle. PCAdmix was assigned with a posterior probability threshold of 0.8. In order to remove highly linked alleles from different populations and avoid spurious ancestry transitions, ancestral populations were thinned using a SNPs pairwise linkage disequilibrium (LD) value (*r*^2^) of <0.8. We defined a constant recombination rate of 1e-8 based on the assumption that 0.01 recombination occur per 1,000 kb (equivalent to 1 cM) (Khayatzadeh et al., 2016).

### Identification of Selection Signatures

To identify signatures of selection we used LD-based methods that search for haplotypes driven to complete fixation (Vitti et al., 2013). These include the integrated haplotype score (iHS), which is a within-population statistic reflecting the amount of extended haplotype homozygosity (EHH) for a given SNP along the ancestral allele relative to the derived allele. Because of the limitation of this statistic when the selected allele is near fixation, we also used the method developed by Tang et al. (2007) that compares EHH profiles between pairs of populations. Based on EHHS, a so-called “site-specific EHH measure,” the Tang et al. method estimates a weighted average of the EHH at both alleles of each SNP in each population. Then, the distribution of the standardized log-ratio of the integrated EHHS (iES) between pairs of populations (referred to as Rsb) is used to detect signals of selection. The advantage of the Tang et al. method is that it calculates EHH for the entire population instead of partitioning it into ancestral and derived alleles, which eliminates the allele frequency constraint and makes it capable of detecting selection sweeps near fixation. The Rsb scores for Simbra crossbred cattle were calculated using the Simmental and Brahman as a reference population.

In this study, the ancestral alleles required for the computation of iHS were inferred as the most common alleles in the entire dataset following Bahbahani and Hanotte (2015). Haplotypes for the iHS and Rsb analyses were derived with fastPHASE (Scheet and Stephens, 2006) using 10 starts (T10) and 25 iterations (C25) of the expectation-maximization (EM) algorithm (Scheet and Stephens, 2006). The iHS and Rsb analyses were performed using the rehh package (Gautier and Vitalis, 2012) in R version 3.4.4. For the analysis of within-population an iHS score >5 (equivalent to *P*-value = 1e-06) and for the analysis of between-population differences a Rsb score >5 (equivalent to *P*-value = 1e-06) were used to infer the candidate genomic regions under selection.

We also examined the gene content within genomic regions containing signatures of selection. This was done using the annotated UMD3.1 reference genome for the Taurine breed Hereford available on the Bovine Genome Database (https://bovinegenome.elsiklab.missouri.edu/). To determine potential overlap of these regions with previously published quantitative trait loci (QTLs), the bovine database (http://www.animalgenome.org/cgi-bin/QTLdb/BT/search) incorporated in the Animal QTL database (Animal QTLdb) of Hu et al. (2019), was used.

## Results

### SNP Genotyping and Quality Control

**Figure 2:**
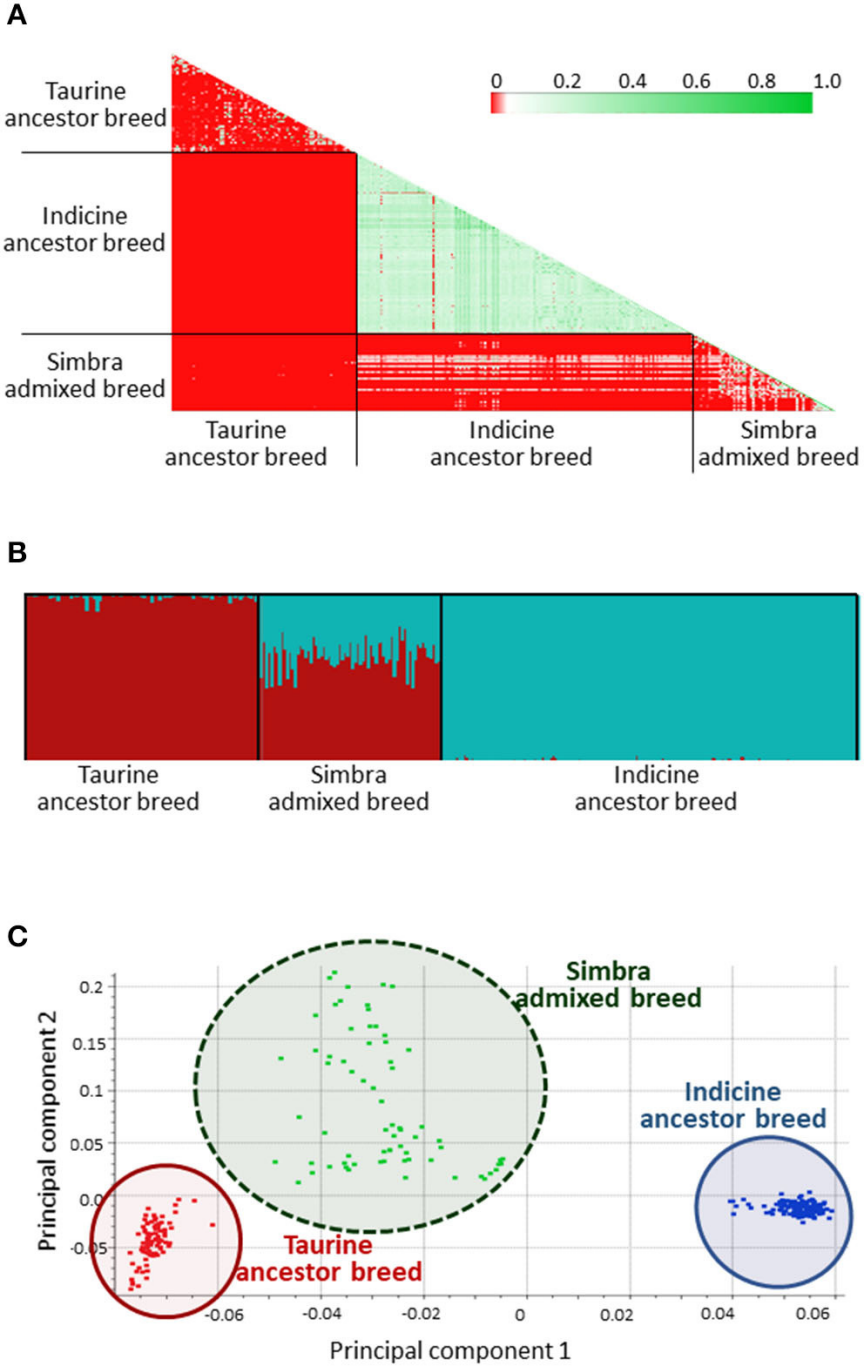
Identity-by-descent (IBD) results of the crossbred South African Simbra population, as well as the ancestral South African Simmental and Brahman populations **(A)**. Green indication a closer genetic distance and red indicating that the genetic distance is farther. FastSTRUCTURE (Raj et al., 2014) results from the 7k SNP panel set at *K* = 2 according to the historical number of ancestral populations (Smith, 2010). Simmental ancestry are indicated in red, while Brahman ancestry are indicated in blue **(B)**. First principal component (PC1) vs. second principal component (PC2) results from the principal component analysis (PCA) for the Simbra, Simmental and Brahman populations computed using the SNP and Variant Suite v.8.8.3 (Golden Helix Inc., Bozeman, MT, USA; www.goldenhelix.com) **(C)**.

After quality control to remove SNPs with <95% call rate, MAF <0.05 and LD (*r*^2^ = 0.8), 4 488 SNPs were retained for analyses. We also performed a sample filtering to limit the inclusion of very closely related individuals (Figure 2A). Accordingly, all 321 animals were retained for analysis (i.e., 69 Simbra, 161 Brahman, and 91 Simmental genomes), based on IBD values of ≥0.45. IBD represents the probability that two randomly chosen alleles of an individual are inherited from a common ancestor, with the length of haplotypes shared between individuals being inversely proportional to the time since divergence from that common ancestor (Browning and Browning, 2010).

### Genetic Diversity

Among the three populations, Simbra and Simmental had comparable observed heterozygosity values (i.e., 0.427 with standard deviation [±SD] of 0.020 and 0.417 with ±SD 0.015, respectively), which were much higher than those for Brahman (0.295, ±SD 0.029, *n* = 161). In comparison with the Simmental (0.0003, ±SD 0.031) and Simbra cattle (−0.011, ±SD 0.045) populations, limited diversity was observed for Brahman (0.022, ±SD 0.103) population.

### Simbra Population Structure and Genomic Content

FastSTRUCTURE separated the animals genotyped in this study into three distinct clusters (Figure 2B). A similar clustering pattern was observed using PCA (Figure 2C), where 55.66% of the genetic variability was explained by the first two principal components (with the first explaining 50.2%). These three clusters corresponded to the Brahman and Simmental ancestor populations, and the Simbra population, representing an admixture between the Taurine and Indicine cattle.

**Figure 3:**
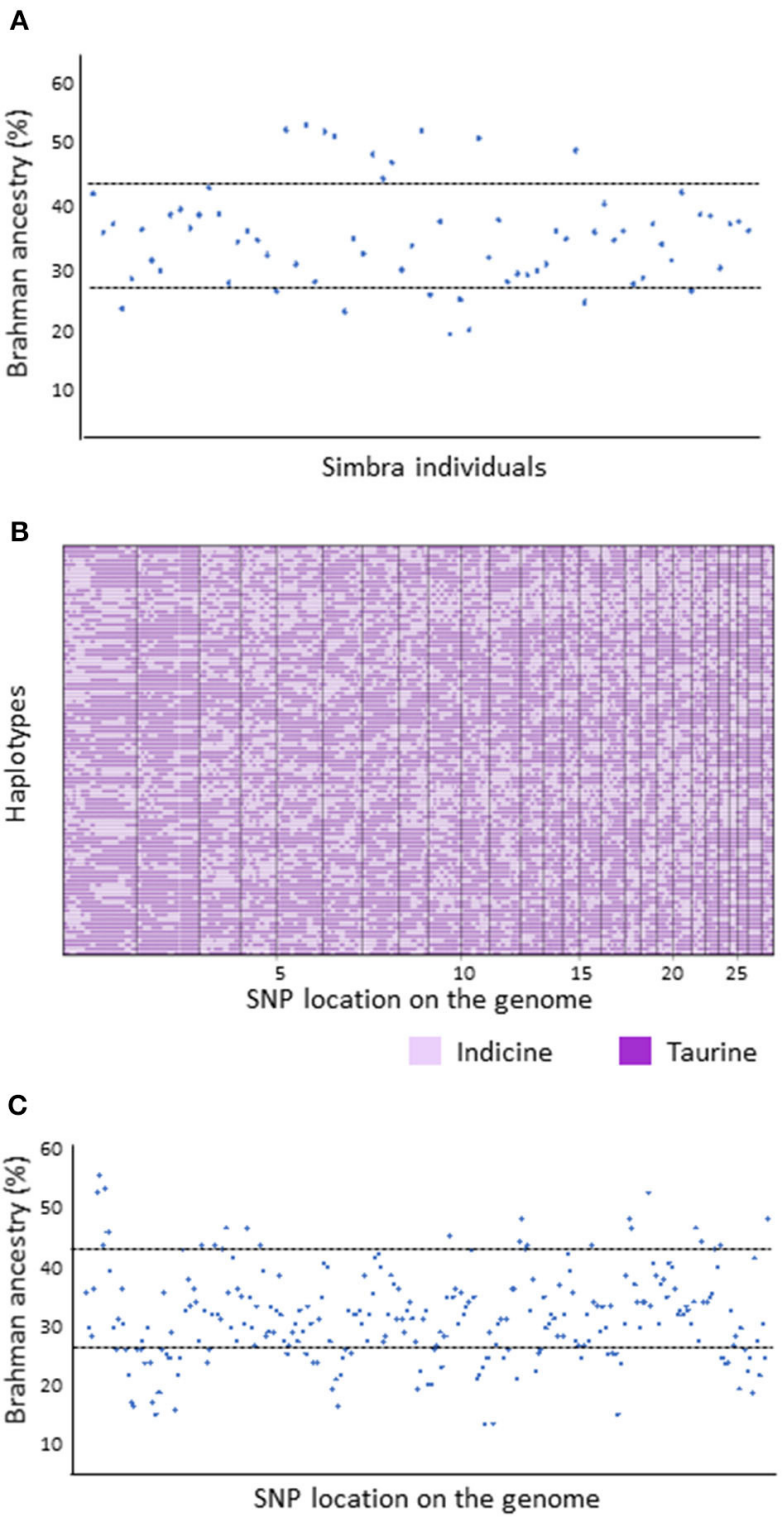
Local ancestry for the crossbred South African Simbra cattle population **(A)** and representative haplotypes **(B,C)** inferred using PCAdmix (Brisbin et al., 2012). The Brahman and Simmental cattle populations were used as source populations (Smith, 2010).

The Simbra hybrid genomes were partitioned into segments of inferred Simmental and Brahman ancestry using the PCAdmix algorithm (Figure 3). We used the default parameters in PCAdmix thereby removing SNPs in high LD (*r*^2^ > 0.8) and SNPs that were monomorphic between the breeds. Subsequent ancestry inference of each genome revealed that the South African Simbra breed is composed of a higher average proportion of Taurine (64.8%, ±SD 8) than Indicine (35.2%, ±SD 8) backgrounds (Figure 3A), as was expected for the breed (O' Connor et al., 1997; Smith, 2010). However, 19 of the 69 Simbra individuals had genomic compositions that deviated substantially from this expectation (Figure 3A); i.e., the Indicine contribution was <27.2% in 9 genomes and >43.2% in 10 genomes.

Using the PCAdmix algorithm, we determined the most probable ancestry along each chromosome of the Simbra genomes (Figures 3B,C). Accordingly, we identified 256 genetic ancestry blocks (i.e., block SNPs with the same inferred ancestry), spread across 29 *Bos taurus* autosomes (BTA1–BTA29) with polymorphic SNPs (call rate less <95% and MAF >5% across all breeds). Of these blocks, 191 (75%) showed a similar pattern as observed above for the average genome composition (i.e., 64.8%, ±SD 8 with Taurine and 35.2%, ±SD 8 with Indicine). The remaining 65 deviated substantially from the expected distribution pattern, with 22 blocks (33.9%) having an excess of Indicine ancestry blocks (>43.2% Brahman blocks) and 43 blocks (66.1%) having excess Taurine ancestry (>27.2% Simmental blocks).

### Genomic Regions Containing Signatures of Positive Selection

**Figure 4:**
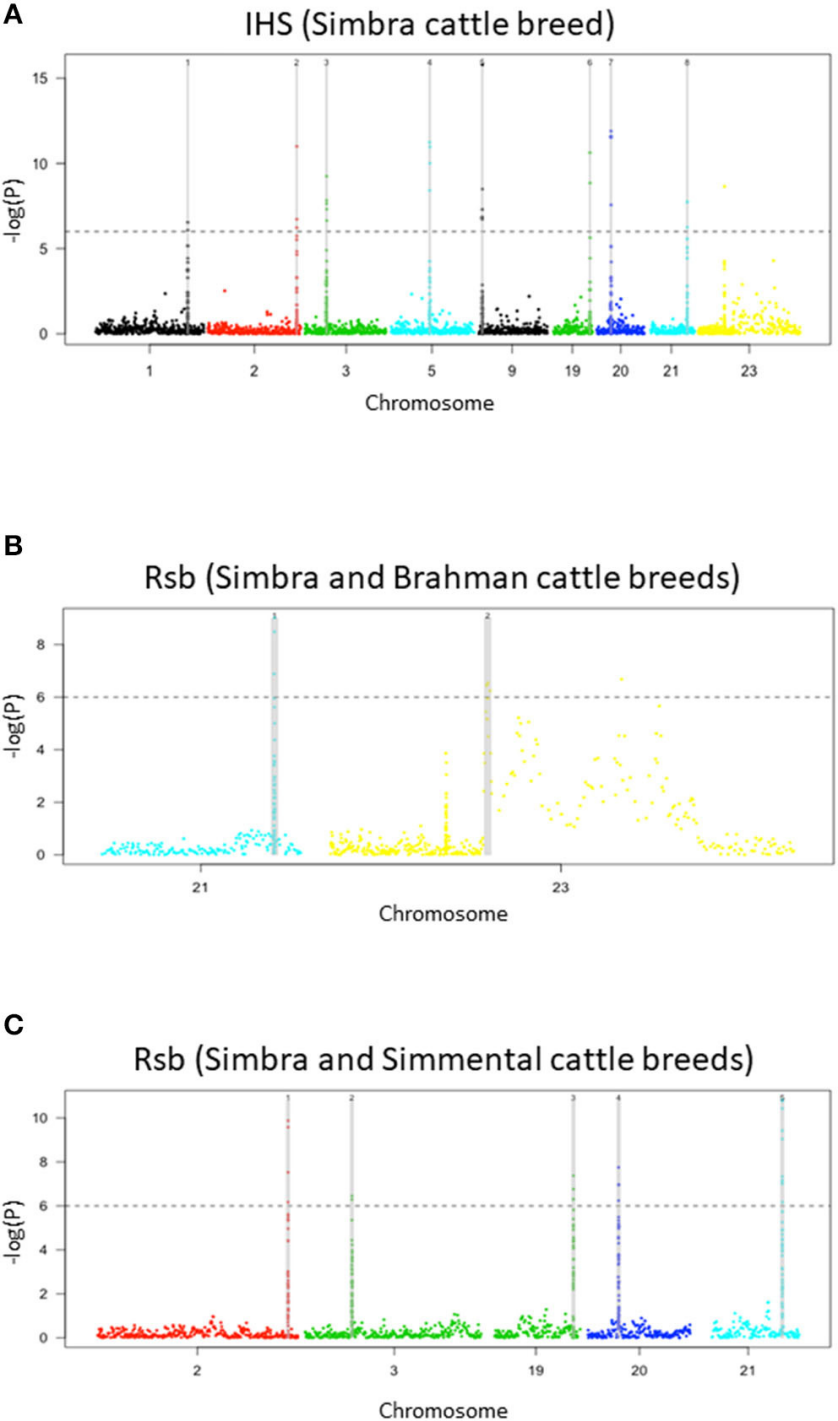
Manhattan plots of genome-wide signatures of positive selection analyses. Distribution of *iHS* scores in the Simbra crossbred cattle **(A)**, *Rsb* analysis with the Simbra and Simmental cattle **(B)**, and *Rsb* analysis with the Simbra and Brahman cattle **(C)**. The iHS and Rsb analysis was performed using the rehh package (Gautier and Vitalis, 2012) in R v. 3.4.4. The dashed line corresponds to a significance threshold (–log_10_) that was set at 6, which is equivalent to *P*-value = 1e−06.

Our analyses revealed the presence of nine genomic regions containing signatures of positive selection in the Simbra genome (Table 1). These regions were identified using intra-population iHS and inter-population Rsb analyses (Vitti et al., 2013). Focusing on the Simbra hybrid cattle, the intra-population iHS analysis identified eight of these regions, which were located on BTA 1, BTA 2, BTA 3, BTA 9, BTA 19, BTA 20, and BTA 21 (Table 2; Figure 4A). Additionally, the Rsb analyses identified five positive selection regions (i.e., on BTA 2, BTA 3, BTA 19, BTA 20, and BTA 21) using Simmental as reference population, and two using Brahman as reference population (i.e., on BTA 21 and on BTA 23) (Table 2; Figures 4B,C). Five of these genomic regions were detected using both the iHS and Rsb statistics. The region on BTA 21 was identified with Rsb analyses employing both Simmental and Brahman as reference populations, while the remainder (i.e., on BTA 2, BTA 3, BTA 19, and BTA 20) were detected using the Simmental reference population. Overall, five (BTA 1, BTA 3, BTA 5, BTA 21, and BTA 23) of the nine regions in which positive selection was detected were located within genetic ancestry blocks that displayed a deviation in the expected genomic composition for Simbra (Table 2).

**Table 1:** Genomic regions identified using iHS and Rsb being under divergent selection in Simbra crossbred cattle and Brahman and Simmental as reference breeds.

**Selection test^a^**	**Selected population**	**Selection region position (Mb)^b^**	**Statistic scores^c^**	**Selective sweepregion size (Mb)**	**Number of SNPs**	**Number of genes**	**Ancestry deviation^d^**
iHS	Simbra	BTA1:131.6–133.5	6.54	1.9	49	13	Taurine excess
iHS	Simbra	BTA2:126.6–128.5	11.00	1.9	51	44	-
Rsb	Simbra+Simmental	BTA2:126.6–128.6	10.46	2	51	44	-
iHS	Simbra	BTA3:32.0–34.0	9.25	2	88	34	Taurine excess
Rsb	Simbra+Simmental	BTA3:32.0–33.9	6.62	1.9	99	34	Taurine excess
iHS	Simbra	BTA5:55.6–57.7	11.24	2.1	66	37	Indicine excess
iHS	Simbra	BTA9:9.8–11.8	15.77	2	76	33	-
iHS	Simbra	BTA19:55.7–57.7	10.6	2	56	77	-
Rsb	Simbra+Simmental	BTA19:55.6–57.7	5.7	2.1	55	77	-
iHS	Simbra	BTA20:21.2–23.2	11.89	2	81	6	-
Rsb	Simbra+Simmental	BTA20:21.2–23.2	5.22	2	90	6	-
iHS	Simbra	BTA21:56.6–58.7	7.76	2.1	56	20	Indicine excess
Rsb	Simbra+Brahman	BTA21:56.8–58.7	5.79	1.9	56	20	Indicine excess
Rsb	Simbra+Simmental	BTA21:56.8–58.7	6.05	1.9	55	20	Indicine excess
Rsb	Simbra+Brahman	BTA23:38.3–40.3	5.13	2.0	14	11	-

a *Signatures of selection was identified using the two LD-based methods (Rsb and iHS) (Vitti et al., 2013)*.

b *Candidate regions are represented as (BTA: start – stop Mb), BTA, Bos taurus autosomes*.

c *Rsb and IHS score >5 (equivalent to P-value = 1e−05) were used to infer the candidate genomic regions under selection*.

d *Regions that displayed a deviation in the expected genomic composition for Simbra, with either an excess of Indicine or Taurine ancestry*.

**Table 2:** Functional annotation of genomic regions showing evidence of selection in the Simbra crossbred cattle.

**Selection test^a^**	**Selected population**	**Selection region position (Mb)^b^**	**Top significant SNP^c^**	**QTL^d^**	**Biological role^e^**	**References**
iHS	Simbra	BTA1:131.6–133.5	BovineHD0100037757	170016	Interval to first estrus after calving	Zhang et al., 2019
iHS, Rsb	Simbra, Simbra+Simmental	BTA2:126.6–128.6	BovineHD0200037032	125219	Lactation persistency	Do et al., 2014
iHS	Simbra	BTA3:32.0–34.0	BovineHD0300010276	179821	Ketosis	Nayeri et al., 2019
iHS	Simbra	BTA5:55.6–57.7	BovineHD0500016044	10570	Ovulation rate	Kirkpatrick et al., 2000
iHS	Simbra	BTA9:9.8–11.8	BovineHD0900002705	15914	Carcass weight	Berkowicz et al., 2012
iHS, Rsb	Simbra, Simbra+Simmental	BTA19:55.6-57.7	BovineHD1900016000	4383	Residual feed intake	Berkowicz et al., 2012
iHS, Rsb	Simbra, Simbra+Simmental	BTA20:21.2–23.2	BovineHD2000006648	5016	Heat intensity	Hoglund et al., 2009
iHS, Rsb	Simbra, Simbra+Simmental, Simbra+Brahman	BTA21:56.6–58.6	BovineHD2100016574	172178	Milk lauric acid content	Gebreyesus et al., 2019
Rsb	Simbra+Brahman	BTA23:38.3–40.3	BovineHD2300011367	11177	Body weight (birth)	McClure et al., 2010

a *Signatures of selection was identified using the two LD-based methods (Rsb and iHS) (Vitti et al., 2013)*.

b *Candidate regions are represented as (BTA: start – stop Mb), BTA, Bos taurus autosomes*.

c *Top significant SNP for the Rsb and iHS analyses*.

d *Potential overlap of the regions that display signatures of selection with previously published quantitative trait loci (QTLs) in the bovine database (http://www.animalgenome.org/cgi-bin/QTLdb/BT/search)*.

e *Biological role of the QTL in the bovine database (http://www.animalgenome.org/cgi-bin/QTLdb/BT/search)*.

Comparison of all of the identified genomic regions harboring signals for positive selection signatures to the genomic resources included in the Animal QTL database, indicated that nine of the identified regions overlapped with those underlying previously published QTLs for cattle (Table 2). These QTLs were previously linked to different biological properties, including reproduction (interval to first oestrus after calving, QTL:170016; lactation persistency, QTL:125219; ovulation rate, QTL:10570), milk traits (milk lauric acid content, QTL:172178), production traits (residual feed intake, QTL:4383; carcass weight, QTL:15914), health (ketosis, QTL:179821), and adaptation traits (Heat tolerance, QTL:31195).

The candidate genomic regions with signatures of positive selection also harbored annotated genes (6–77 genes) (Tables 1, 3, Supplementary Table 1). These included genes that encode putative kinesin family member 13A (KIF13A), the small integral membrane protein 5 (SMIM5), MIER family member 3 (MIER3), Solute carrier family 24 member 4 (SLC24A4), muscle-specific ligases tripartite motif containing 63 (TRIM63; also called muscle-specific ring-finger protein 1 or MuRF-1), as well as the potassium voltage-gated channel subfamily A member (KCNA10).

**Table 3:** Examples of candidate genes within the candidate regions of the different analyses conducted in the study.

**Selected population^a^**	**Selection region position (Mb)^b^**	**ENSEMBLE gene ID^c^**	**ENSEMBLE Gene name^d^**
Simbra	1:131.6–133.5	ENSBTAG00000014589,ENSBTAG00000008299	Claudin 18 (CLDN18) Interleukin 20 receptor subunit beta (IL20RB)
Simbra, Simbra+Simmental	2:126.6–128.6	ENSBTAG00000001513,ENSBTAG00000005085	PDLIM1 interacting kinase 1 like (PDIK1L) Tripartite motif containing 63 **(**TRIM63)
Simbra	3:32.0–34.0	ENSBTAG00000015459	Potassium voltage-gated channel subfamily A member 2 (KCNA2)
Simbra	5:55.6–57.7	ENSBTAG00000018361, ENSBTAG00000008322	R3H domain containing 2 (R3HDM2) Potassium voltage-gated channel subfamily A member 10 **(**KCNA10)
Simbra	9:9.8–11.8	ENSBTAG00000048046	Uncharacterized protein
Simbra, Simbra+Simmental	19:55.6–57.7	ENSBTAG00000010758,ENSBTAG00000011713	Small integral membrane protein 5 **(**SMIM5) Uncharacterized protein
Simbra, Simbra+Simmental	20:21.2–23.2	ENSBTAG00000014248, ENSBTAG00000047548	MIER family member 3 Uncharacterized protein (MIER3)
Simbra, Simbra+Simmental, Simbra+Brahman	21:56.6–58.6	ENSBTAG00000006620	Solute carrier family 24 member 4 (SLC24A4)
Simbra+ Brahman	23:38.3–40.3	ENSBTAG00000019217	Kinesin family member 13A (KIF13A)

a *Signatures of selection was identified using the two LD-based methods (Rsb and iHS) (Vitti et al., 2013)*.

b *Candidate regions are represented as (BTA: start – stop Mb), BTA, Bos taurus autosomes*.

c *ENSEMBLE gene ID obtained from Ensembl (http://www.ensembl.org/index.html)*.

d *ENSEMBLE gene name obtained from Ensembl (http://www.ensembl.org/index.html)*.

## Discussion

This is the first study to utilize genome-wide polymorphism data to investigate the genetic diversity, population structure and patterns of local ancestry of the South African Simbra hybrid breed and its Taurine and Indicine ancestor breeds. We also used the SNP data obtained to identify candidate genomic regions with signatures of adaptive introgression and positive selection. The availability of the genome sequencing data from the SA Beef Genomics Project will make it possible in the future to augment conventional livestock breeding and performance management programmes with genomic information.

Our results showed that hybridization of the Taurine and Indicine breeds conferred a higher genetic diversity of the Simbra breed in comparison with the purebred breeds (Ghafouri-Kesbi, 2010; Zhang et al., 2015). This was obvious from the negative inbreeding coefficient (*f*) estimate that indicated an excess of heterozygosity even beyond what is expected under Hardy-Weinberg equilibrium in the Simbra population (Maiorano et al., 2018). Compared to the two ancestral breeds, the South African Simbra population had the highest genetic diversity, although it was only marginally higher than that of the Simmental breed. Therefore, hybridization of subspecies remains an important tool for expanding the genetic variation within modern cattle breeds (Gregory and Cundiff, 1980). Also, the genetic diversity inherent to South African Simbra holds significant potential for improvements in production and environmental adaptability (Sölkner et al., 1998; Becker et al., 2013).

The limited diversity observed for Brahman breed is most likely a consequence of intensive artificial selection for improved productivity (Albertí et al., 2008). It was previously suggested that the low genetic diversity in the Brahman breed may be partly ascribed to the use of elite sires (Makina et al., 2014). Such practices are consistent with the observed F value (0.0003), which are suggestive of some inbreeding in the Brahman populations examined (van der Westhuizen et al., 2019). Genetic diversity within the Simmental population was slightly higher than in the Brahman breed. This may be because the cattle BeadChip was optimized for use in *Bos taurus taurus* breeds (Cheruiyot et al., 2018).

Genome-wide polymorphism data indicated that the genomic background of the South African Simbra hybrid breed represents a mosaic of the Taurine and Indicine ancestor breeds, as was expected (Smith, 2010). Our data also confirmed the optimal 5/8 Simmental and 3/8 Brahman composition of the Simbra genomes included in this study, since this composition ensures maintenance of favorable traits from both breeds (i.e., meat tenderness of the Simmental breed and heat-tolerance of the Brahman breed) (O' Connor et al., 1997; Smith, 2010). Additionally, the PCA and FastSTRUCTURE data also clearly demonstrated that the South African Simbra has evolved into a unique breed, as three distinct clusters were identified. This suggests that, after initial formation and subsequent intense artificial selection and breeding, the Simbra breed composition has stabilized over time (Paim et al., 2020).

Our results suggested that crossbreeding, followed by selection, was key in shaping the genome of the South African Simbra hybrid breed (Ríos-Utrera et al., 2020). Consistent with previous studies (e.g., Bahbahani and Hanotte, 2015; Bahbahani et al., 2017), the two EHH-based statistics used in this study allowed for the identification of genomic regions that display signatures of positive selection in the hybrid genome. These included regions that were identified using the intra-population iHS statistics, as well as the inter-population Rsb statistics using the Simmental and Brahman cattle as reference populations. The candidate regions identified using the iHS and Rsb statistics supports the role of selection pressures, and not natural demographic processes, in shaping the genomic pattern of these regions (Bahbahani et al., 2018). Also, 25% of the regions displayed ancestry deviation. Furthermore, only five genomic regions that displayed signatures of positive selection overlapped with regions containing locus-ancestry deviation. This may be because EHH-based statistics identify older signals of selection, while ancestry deviation is likely caused by recent post-admixture selection (Oleksyk et al., 2010; Bahbahani et al., 2018). Regions that display ancestry deviation observed in the young Simbra crossbreed that was developed in the United States in the late 1960s (Gouws, 2016), is most likely the result of recent post-admixture selection.

The South African Simbra hybrid breed appears to be evolving separately from its ancestoral breeds, with selection driving the increase in prevalence of advantageous alleles derived from both the parent breeds (Xu et al., 2015). The presence of genomic regions displaying locus-ancestry deviation supports the likelihood that they are important for the adaptability of Simbra cattle to the local environment (Bahbahani et al., 2018). The inter-population Rsb statistics, using Brahman as reference, allowed for the identification of Taurine haplotypes in regions that are under selection. Similarly, Rsb statistics using Simmental as reference allowed for the identification of regions that support selection pressures on Indicine haplotypes. As suggested recently, the identified genomic regions under selection may have adaptive significance to maximize their reproductive fitness and their adaptability to environmental challenges (Bahbahani et al., 2018).

Analysis of genes and known QTLs in regions of the Simbra genome that harbor signals of positive selection suggest that these are likely involved in its improved environmental adaptability and productivity (Paim et al., 2020; Ríos-Utrera et al., 2020). Many of the genes located in these genomic regions have previously been implicated in traits that are highly valued in the Simbra composite breed (Smith, 2010). The location of these regions also overlapped or co-occurred with previously reported bovine quantitative trait loci (QTLs) (https://www.animalgenome.org), which strongly reflect the overall breeding goals of the Simbra breed (Smith, 2010). For example, one of the adaptive regions located on BTA 23 co-occurred with a QTL associated with body weight (Lu et al., 2013). This region that is derived from the Simmental ancestry is important for growth performance in the Simbra breed (Pico, 2004; Amen et al., 2007; Smith, 2010; Maúre et al., 2018). The heritability of these traits may be due to positive selection of gene regions that is caused by beneficial polymorphisms in the genes affecting the traits, because mutation that provides a fitness advantage will increase in frequency in the population (Taye et al., 2017).

Most of the genomic regions experiencing positive selection were implicated in traits that are valued in breeds of Indicine ancestry. For example, the region located on BTA 5 that displays locus-ancestry deviation (excess of Brahman parent alleles) co-occurred with a QTL associated with ovulation rate. This confirms that regions/genes related to fertility and reproduction are hotspots of selection in breeds living in tropical environments (Bahbahani et al., 2018). The region located on BTA 20 co-occurred with a QTL associated with heat intensity (i.e., heat tolerance), and is derived from the Brahman ancestry. Adaptation to the harsh South African environment that is valued in the Indicine parent breed will allow for the Simbra breed to adapt to climate change that will likely cause South Africa to become hotter and drier (Girvetz et al., 2019). Of the genomic regions displaying positive selection, and that co-occurred with known QTLs linked with production in the Simmental breed, many were also previously demonstrated to be under selection in Western and Russian Simmental populations (Mészáros et al., 2019). These included QTLs associated with carcass weight that are located on BTA 9, milk production located on BTA 2 and BTA 21, as well as fertility located on BTA 1, that display locus-ancestry deviation (excess of Simmental parent alleles) (Berkowicz et al., 2012; Do et al., 2014; Gebreyesus et al., 2019; Zhang et al., 2019). These genomic regions include genes that encode for a SLC24A4 homolog located on BTA 21, which is known to be associated with milk production and fertility (Nayeri and Stothard, 2016; Nayeri et al., 2016). Our results could therefore highlight new regions and pathways that may contribute to variation in reproductive health, fertility, and milk production in cattle in general.

Many of the genes occurring in regions under positive selection in Simbra were previously identified using genome-wide association studies (GWAS) where they were linked to meat quality of Taurine, Indicine and composite breeds (Allais et al., 2014; Hulsman et al., 2014; Magalhães et al., 2016; Xia et al., 2016). For example, KCNA10 encoded on BTA 3 is likely involved in determining meat quality in Simbra that may be derived from the Simmental parent breed (Lang et al., 2000; Fleet et al., 2011). Other genes, derived from the Brahman parent breed that include SMIM5 encoded on BTA 19 that display locus-ancestry deviation (excess of Brahman parent alleles), may negatively influence carcass and meat properties (e.g., marbling) (Mateescu et al., 2017; Taye et al., 2017). Some of the adaptive alleles identified in Simbra were implicated in the sensory characteristics of meat (e.g., tenderness, flavor, juiciness, and color), which are mainly affected by proteolytic activities of muscle (Taye et al., 2017). For example, a homolog of TRIM63 (also called MuRF-1), located on BTA 2, has been linked with meat tenderness in Nellore cattle (Indicine) (Muniz et al., 2016). MuRF-1 is an important component of the ubiquitin-proteasome system, which is the main proteolytic pathway in skeletal muscle growth in domestic animals (Koohmaraie et al., 2002). This pathway regulates the balance between the amounts of muscle proteins synthesized and degraded to control the skeletal muscle mass (Koohmaraie et al., 2002). Accordingly, the ubiquitin-proteasome system and its components have been linked to meat tenderness (Yin et al., 2010; Taye et al., 2017), productivity and economic value of animals (Sadri et al., 2016; Nakanishi et al., 2019). The high number of genes identified in this study and other studies that are associated with meat quality, underscore the complexity of this trait and that it is regulated by multiple interrelated causative factors and layers of feedback regulation (Diniz et al., 2019).

Some of the genomic regions subject to positive selection are likely involved in overall health and fitness of the Simbra breed. For example, the region located on BTA 3, which is known to be under selection in Western and Russian Simmental populations (Mészáros et al., 2019) and most likely derived from the Simmental parent breed, overlaps with a QTL associated with ketosis (QTL:179821). The latter is a metabolic disorder where negative energy balances (when energy demand exceeds intake) affect animal health and productivity (Nayeri et al., 2019). It has been postulated that such failure to maintain internal homeostatic and homeorhetic regulation maybe caused by intense genetic selection (Nayeri et al., 2019). Furthermore, metabolic disorders have also been demonstrated to negatively influence the immune response in cattle (Wathes et al., 2009; Esposito et al., 2014). The results of this study can be used for further genetic analysis to identify causal variants that affect ketosis and metabolic diseases.

Likewise, health and fitness traits that had likely been derived from Indicine ancestry were also encoded in Simbra genomic regions subject to selection. These regions are located on BTA 5, BTA 19, BTA 20, and BTA 21, which appear to be derived from Brahman. BTA 5 harbors a gene encoding KCNA10 (potassium voltage-gated channel subfamily A member 10) known to influence potassium metabolism and play a role in human and animal production and health (Lang et al., 2000; Fleet et al., 2011). This protein regulates acid-base balance and maintains cellular pH and electrical gradients (Lang et al., 2000; Fleet et al., 2011), which has previously been demonstrated to influence meat quality in cattle (Diniz et al., 2019). Likewise, BTA 21 contains the SLC24A4 gene that encodes a member of potassium-dependent sodium or calcium exchanger protein family, which may influence pigmentation related traits that may influence health (e.g., UV protection) (Sulem et al., 2007). The selection region on BTA 19 contains a gene encoding the small integral membrane protein 5 (SMIM5) that is associated with udder health and clinical mastitis in Holstein cattle (Wu et al., 2015). The region experiencing selection on BTA 20 harbors a gene that encodes MIER family member 3 Uncharacterized protein (MIER3), which is associated with survival in Holstein and Jersey cattle (Raven et al., 2014).

Finally, analysis of genome-wide polymorphisms further showed that the genetic diversity of the South African purebred Brahman parental breed was slightly lower than the Simmental population. This is similar to what has been reported previously (Qu et al., 2006; Agung et al., 2016; Utrera et al., 2018). The low level of diversity in the Brahman breed may be an indication of relative homogeneity in the South African populations as a consequence of intensive artificial selection for improved productivity (Albertí et al., 2008; Taberlet et al., 2008). It was also previously suggested that the low genetic diversity observed in the Brahman breed may be partly ascribed to the use of elite sires (Makina et al., 2014). Such practices are consistent with the observed inbreeding coefficient (*f*) estimate (0.022), which is suggestive of some inbreeding in the Brahman populations examined (van der Westhuizen et al., 2019). Although it cannot be excluded that the low genetic diversity in the Brahman population may be due to the fact that the cattle BeadChip was optimized for use in *Bos taurus taurus* breeds (Cheruiyot et al., 2018), it is important that genetic diversity must be maintained and increased for sustainable production and management of this purebred cattle breed.

## Conclusions

The SNP array data allowed for the assessment of genetic diversity, population structure and admixture of the South African Simbra population. Our findings contribute to the current knowledge of the genetics of the Simbra breed, and provides insight into how genomic architecture changes with hybridization and crossbreed formation. Results of this study emphasize the importance of assessing the genetic diversity, population structure and admixture of other South African cattle breeds. It also emphasize the importance of implementing a management strategy to increase diversity in the purebred breeds.

The genome-wide SNP array further allowed for the identification of signatures of positive selection in the Simbra hybrid genome, and these putatively introgressed genomic regions may have adaptive significance, affecting important phenotypic traits (e.g., adaption, reproduction, and production) in the breed. These include Indicine-derived alleles associated with heat tolerance and Taurine-derived alleles that are associated with body weight.

Knowledge of the genetics controlling meat quality will increase the ability of the industry to produce better meat, which will benefit consumers and should increase the demand for beef, which is of great interest to the beef industry (Mateescu et al., 2017). The identified adaptive introgression of alleles of Indicine- and Taurine derived ancestral genes may lay the foundation for *ad-hoc* physiological studies and targets for selection (and potentially gene editing), that may increase production and health in modern cattle breeds. Ultimately, this study represents an important step toward developing and improving strategies for targeted selection and breeding that will ultimately contribute meaningfully to the beef production industry of South Africa.

## Data Availability Statement

The raw data supporting the conclusions of this article will be made available by the authors, without undue reservation.

## Ethics Statement

The animal study was reviewed and approved by Agricultural Research Council Animal Research Ethics Committee.

## Author Contributions

MN, FM, and MB conceived of the presented idea. MN, LD, and NM developed the theory. MN, NH, KH, and W-YC performed the computations. BG, BK, ED, and PS verified the analytical methods. All authors discussed the results and contributed to the final manuscript.

## Conflict of Interest

The authors declare that the research was conducted in the absence of any commercial or financial relationships that could be construed as a potential conflict of interest.

## References

[B1] Agung P. P. Saputra F. Septian W. A. (2016). Study of genetic diversity among Simmental cross cattle in West Sumatra based on microsatellite markers. Asian-Austral. J. Anim. Sci. 29:176. 10.5713/ajas.15.0155 26732442 PMC4698697

[B2] Ajmone-Marsan P. Garcia J. F. Lenstra J. A. (2010). On the origin of cattle: how aurochs became cattle and colonized the world. Evol. Anthropol. 19, 148–157. 10.1002/evan.20267

[B3] Albertí P. Panea B. Sañudo C. Olleta J. Ripoll G. Ertbjerg P. (2008). Live weight, body size and carcass characteristics of young bulls of fifteen European breeds. Livest. Sci. 114, 19–30. 10.1016/j.livsci.2007.04.010

[B4] Allais S. Levéziel H. Hocquette J. F. Rousset S. Denoyelle C. Journaux L.. (2014). Fine mapping of quantitative trait loci underlying sensory meat quality traits in three French beef cattle breeds. J. Anim. Sci. 92, 4329–4341. 10.2527/jas.2014-7868 25149327

[B5] Amen T. S. Herring A. D. Sanders J. O. Gill C. A. (2007). Evaluation of reciprocal differences in Bos indicus x Bos taurus backcross calves produced through embryo transfer: II. Post weaning, carcass, and meat traits. J. Anim. Sci. 85, 373–379. 10.2527/jas.2005-755 17235022

[B6] Bahbahani H. Afana A. Wragg D. (2018). Genomic signatures of adaptive introgression and environmental adaptation in the Sheko cattle of southwest Ethiopia. PLoS ONE 13:e0202479. 10.1371/journal.pone.0202479 30114214 PMC6095569

[B7] Bahbahani H. Hanotte O. (2015). Genetic resistance: tolerance to vector-borne diseases, prospect and challenges of genomics. OIE Sci. Tech. Rev. 34, 185–197. 10.20506/rst.34.1.2353 26470457

[B8] Bahbahani H. Tijjani A. Mukasa C. Wragg D. Almathen F. Nash O.. (2017). Signature of selection for environmental adaptation and zebu x taurine hybrid fitness in East African Shorthorn Zebu. Front. Genet. 8:68. 10.3389/fgene.2017.00068 28642786 PMC5462927

[B9] Becker M. Gruenheit N. Steel M. Voelckel C. Deusch O. Heenan P. B. (2013). Hybridization may facilitate in situ survival of endemic species through periods of climate change. Nat. Clim. Change 3, 1039–1043. 10.1038/nclimate2027

[B10] Bennett G. L. Gregory K. E. (1996). Genetic (co) variances among birth weight, 200-day weight, and postweaning gain in composites and parental breeds of beef cattle. J. Anim. Sci. 74, 2598–2611. 10.2527/1996.74112598x 8923174

[B11] Berkowicz E. W. Magee D. A. Berry D. P. Sikora K. M. Howard D. J. Mullen M. P.. (2012). Single nucleotide polymorphisms in the imprinted bovine insulin-like growth factor 2 receptor gene (IGF2R) are associated with body size traits in Irish Holstein-Friesian cattle. Anim. Genet. 43, 81–87. 10.1111/j.1365-2052.2011.02211.x 22221028

[B12] Bhati M. Kadri N. K. Crysnanto D. Pausch H. (2020). Assessing genomic diversity and signatures of selection in Original Braunvieh cattle using whole-genome sequencing data. BMC Genom. 21, 1–14. 10.1186/s12864-020-6446-y 31914939 PMC6950892

[B13] Bolormaa S. Porto Neto L. R. Zhang Y. D. Bunch R. J. Harrison B. E. Goddard M. E.. (2011). A genome-wide association study of meat and carcass traits in Australian cattle. J. Anim. Sci. 89, 2297–2309. 10.2527/jas.2010-3138 21421834

[B14] Bordbar F. Jensen J. Du M. Abied A. Guo W. Xu L.. (2020). Identification and validation of a novel candidate gene regulating net meat weight in Simmental beef cattle based on imputed next-generation sequencing. Cell Prolif. 53:e12870. 10.1111/cpr.12870 32722873 PMC7507581

[B15] Bradley D. G. MacHugh D. E. Cunningham P. Loftus R. T. (1996). Mitochondrial diversity and the origins of African and European cattle. PNAS 93, 5131–5135. 10.1073/pnas.93.10.5131 8643540 PMC39419

[B16] Brisbin A. Bryc K. Byrnes J. Zakharia F. Omberg L. Degenhardt J. (2012). PCAdmix: principal components-based assignment of ancestry along each chromosome in individuals with admixed ancestry from two or more populations. Hum. Biol. 844, 343–364. 10.3378/027.084.0401 PMC3740525 23249312

[B17] Browning B. L. Zhou Y. Browning S. R. (2018). A one-penny imputed genome from next generation reference panels. Am. J. Hum. Genet. 103, 338–348. 10.1016/j.ajhg.2018.07.015 30100085 PMC6128308

[B18] Browning S. R. Browning B. L. (2010). High-resolution detection of identity by descent in unrelated individuals. Am. J. Hum. Genet. 86, 526–539. 10.1016/j.ajhg.2010.02.021 20303063 PMC2850444

[B19] Cheruiyot E. K. Bett R. C. Amimo J. O. Zhang Y. Mrode R. Mujibi F. D. (2018). Signatures of selection in admixed dairy cattle in Tanzania. Front. Genet. 9:607. 10.3389/fgene.2018.00607 30619449 PMC6305962

[B20] Crouse J. D. Cundiff R. M. Koch M. R. Koohmaraie M. Seideman S. C. (1989). Comparisons of *Bos indicus* and *Bos taurus* inheritance for carcass beef characteristics and meat palatability. J. Anim. Sci. 67, 2661–2668. 10.2527/jas1989.67102661x22054614

[B21] Daetwyler H. D. Capitan A. Pausch H. Stothard P. van Binsbergen R. Brøndum R. F.. (2014). Whole-genome sequencing of 234 bulls facilitates mapping of monogenic and complex traits in cattle. Nat. Genet. 46, 858–865. 10.1038/ng.3034 25017103

[B22] Dikmen S. Mateescu R. G. Elzo M. A. Hansen P. J. (2018). Determination of the optimum contribution of Brahman genetics in an Angus-Brahman multibreed herd for regulation of body temperature during hot weather. J. Anim. Sci. 96, 2175–2183. 10.1093/jas/sky133 29741636 PMC6095375

[B23] Diniz W. J. D. S. Banerjee P. Regitano L. C. (2019). Cross talk between mineral metabolism and meat quality: a systems biology overview. Physiol. Genom. 51, 529–538. 10.1152/physiolgenomics.00072.2019 31545932

[B24] Do C. Waples R. S. Peel D. Macbeth G. M. Tillett B. J. Ovenden J. R. (2014). NeEstimator v2: re-implementation of software for the estimation of contemporary effective population size (Ne) from genetic data. Mol. Ecol. Resour. 14, 209–214. 10.1111/1755-0998.12157 23992227

[B25] Esposito G. Irons P. C. Webb E. C. Chapwanya A. (2014). Interactions between negative energy balance, metabolic diseases, uterine health and immune response in transition dairy cows. Anim. Reprod. Sci. 144, 60–71. 10.1016/j.anireprosci.2013.11.007 24378117

[B26] Fleet J. C. Replogle R. Salt D. E. (2011). Systems genetics of mineral metabolism. J. Nutr. 141, 520–525. 10.3945/jn.110.128736 21270371 PMC3040909

[B27] Frankham R. (2015). Genetic rescue of small inbred populations: meta-analysis reveals large and consistent benefits of gene flow. Mol. Ecol. 24, 2610–2618. 10.1111/mec.13139 25740414

[B28] Gautier M. Vitalis R. (2012). REHH: an R package to detect footprints of selection in genome-wide SNP data from haplotype structure. Bioinformatics 28, 1176–1177. 10.1093/bioinformatics/bts115 22402612

[B29] Gebreyesus G. Buitenhuis A. J. Poulsen N. A. Visker M. H. P. W. Zhang Q. Van Valenberg H. J. F.. (2019). Multi-population GWAS and enrichment analyses reveal novel genomic regions and promising candidate genes underlying bovine milk fatty acid composition. BMC Genomics 20:178. 10.1186/s12864-019-5573-9 30841852 PMC6404302

[B30] Ghafouri-Kesbi F. (2010). Change in genetic size of small-closed populations: lessons from a domestic mammal population. Genet. Mol. Biol. 33, 657–662. 10.1590/S1415-47572010000400011 21637574 PMC3036146

[B31] Girvetz E. Ramirez-Villegas J. Claessens L. Lamanna C. Navarro-Racines C. Nowak A. (2019). Future climate projections in Africa: where are we headed? in The Climate-Smart Agriculture Papers, eds Rosenstock T. S. Nowak A. Girvetz E. (Springer, Cham), 15–27. 10.1007/978-3-319-92798-5_2

[B32] Gouws A. (2016). Genetic ability is more important than feed: on the farm. Stockfarm 6, 20–21.

[B33] Gouws A. (2017). A sought-after breed for crossbreeding. Stockfarm 7, 9–11.

[B34] Gregory K. E. Cundiff L. (1980). Crossbreeding in beef cattle: evaluation of systems. J. Anim. Sci. 51, 1224–1242. 10.2527/jas1980.5151224x

[B35] Greyling B. J. Kryger P. du Plessis S. Van Hooft W. F. Van Helden P. Getz W. M. (2008). Development of a highthroughput microsatellite typing approach for forensic and population genetic analysis of wild and domestic African Bovini. Afr. J. Biotechnol. 7, 655–660.

[B36] Gutiérrez-Gil B. Wiener P. Nute G. R. Burton D. Gill J. L. Wood J. D.. (2008). Detection of quantitative trait loci for meat quality traits in cattle. Anim. Genet. 39, 51–61. 10.1111/j.1365-2052.2007.01682.x 18254735

[B37] Harrison R. G. Larson E. L. (2014). Hybridization, introgression, and the nature of species boundaries. J. Hered. 105, 795–809. 10.1093/jhered/esu033 25149255

[B38] Hoglund J. K. Guldbrandtsen B. Su G. Thomsen B. Lund M. S. (2009). Genome scan detects quantitative trait loci affecting female fertility traits in Danish and Swedish Holstein cattle. J. Dairy Sci. 92, 2136–2143. 10.3168/jds.2008-1104 19389971

[B39] Hu Z. L. Park C. A. Reecy J. M. (2019). Building a livestock genetic and genomic information knowledgebase through integrative developments of Animal QTLdb and CorrDB. Nucleic Acids Res. 47, D701–D710. 10.1093/nar/gky1084 30407520 PMC6323967

[B40] Hulsman H. L. L. Garrick D. J. Gill C. A. Herring A. D. Riggs P. K. Miller R. K. (2014). Genome-wide association study of temperament and tenderness using different Bayesian approaches in a Nellore-Angus crossbred population. Livest. Sci. 161, 17–27. 10.1016/j.livsci.2013.12.012

[B41] Iso-Touru T. Tapio M. Vilkki J. Kiseleva T. Ammosov I. Ivanova Z.. (2016). Genetic diversity and genomic signatures of selection among cattle breeds from Siberia, eastern and northern Europe. Anim. Genet. 47, 647–657. 10.1111/age.12473 27629771

[B42] Johnson D. D. Huffman R. D. Williams S. E. Hargrove D. D. (1990). Effects of percentage Brahman and Angus breeding, age-season of feeding and slaughter end point on meat palatability and muscle characteristics. J. Anim. Sci. 68, 1980–1986. 10.2527/1990.6871980x 2384388

[B43] Khayatzadeh N. Mészáros G. Utsunomiya Y. T. Garcia J. F. Schnyder U. Gredler B. (2016). Locus-specific ancestry to detect recent response to selection in admixed Swiss Fleckvieh cattle. Anim. Genet. 47, 637–646. 10.1111/age.12470 27435758

[B44] Kim J. Hanotte O. Mwai O. A. Dessie T. Bashir S. Diallo B.. (2017). The genome landscape of indigenous African cattle. Genome Biol. 18:34. 10.1186/s13059-017-1153-y 28219390 PMC5319050

[B45] Kirkpatrick B. W. Byla B. M. Gand regory K. E. (2000). Mapping quantitative trait loci for bovine ovulation rate. Mamm. Genome 11, 136–139. 10.1007/s003350010026 10656928

[B46] Koohmaraie M. Kent M. P. Shackelford S. D. Veiseth E. Wheeler T. L. (2002). Meat tenderness and muscle growth: is there any relationship? Meat Sci. 62, 345–352. 10.1016/S0309-1740(02)00127-4 22061610

[B47] Kristensen T. N. Hoffmann A. A. Pertoldi C. Stronen A. V. (2015). What can livestock breeders learn from conservation genetics and vice versa? Front. Genet. 6:38. 10.3389/fgene.2015.00038 25713584 PMC4322732

[B48] Lang R. Lee G. Liu W. Tian S. Rafi H. Orias M.. (2000). KCNA10: a novel ion channel functionally related to both voltagegated potassium and CNG cation channels. Am. J. Physiol - Renal Physiol. 278, F1013–1021. 10.1152/ajprenal.2000.278.6.F1013 10836990

[B49] Loftus R. T. MacHugh D. E. Bradley D. G. Sharp P. M. Cunningham P. (1994). Evidence for two independent domestications of cattle. PNAS 91, 2757–2761. 10.1073/pnas.91.7.2757 8146187 PMC43449

[B50] Low W. Y. Tearle R. Liu R. Koren S. Rhie A. Bickhart D. M.. (2020). Haplotype-resolved genomes provide insights into structural variation and gene content in Angus and Brahman cattle. Nat. Commun. 11, 1–14. 10.1038/s41467-020-15848-y 32350247 PMC7190621

[B51] Lu D. Sargolzaei M. Kelly M. Van der Voort G. Wang Z. Mandell I.. (2013). Genome-wide association analyses for carcass quality in crossbred beef cattle. BMC Genet. 14:80. 10.1186/1471-2156-14-80 24024930 PMC3827924

[B52] Magalhães A. F. B. de Camargo G. M. F. Fernandes G. A. Gordo D. G. M. Tonussi R. L. Costa R. B.. (2016). Genome-wide association study of meat quality traits in nellore cattle. PLoS ONE 11:e0157845. 10.1371/journal.pone.0157845 27359122 PMC4928802

[B53] Maiorano A. M. Lourenco D. L. Tsuruta S. Ospina A. M. T. Stafuzza N. B. Masuda Y.. (2018). Assessing genetic architecture and signatures of selection of dual purpose Gir cattle populations using genomic information. PLoS ONE 13:e0200694. 10.1371/journal.pone.0200694 30071036 PMC6071998

[B54] Makina S. O. Muchadeyi F. C. van Marle-Köster E. MacNeil M. D. Maiwashe A. (2014). Genetic diversity and population structure among six cattle breeds in South Africa using a whole genome SNP panel. Front. Genet. 5:333. 10.3389/fgene.2014.00333 25295053 PMC4170099

[B55] Martínez G. Galíndez R. (2006). Genetic and environmental trends in birth and weaning weights in registered Brahman cattle, in Proceedings of the 8th World Congress on Genetics Applied to Livestock Production (Belo Horizonte: Instituto Prociência), 3–60.

[B56] Mateescu R. G. Garrick D. J. Reecy J. M. (2017). Network analysis reveals putative genes affecting meat quality in Angus cattle. Front. Genet. 8:171. 10.3389/fgene.2017.00171 29163638 PMC5681485

[B57] Maúre G. Pinto I. Ndebele-Murisa M. Muthige M. Lennard C. Nikulin G. (2018). The southern African climate under 1.5°C and 2°C of global warming as simulated by CORDEX regional climate models. Environ. Res. Lett. 13:065002 10.1088/1748-9326/aab190

[B58] McClure M. C. Morsci N. S. Schnabel R. D. Kim J. W. Yao P. Rolf M. M.. (2010). A genome scan for quantitative trait loci influencing carcass, post-natal growth and reproductive traits in commercial Angus cattle. Anim. Genet. 41, 597–607. 10.1111/j.1365-2052.2010.02063.x 20477797

[B59] McClure M. C. Ramey H. R. Rolf M. M. McKay S. D. Decker J. E. Chapple R. H.. (2012). Genome-wide association analysis for quantitative trait loci influencing Warner-Bratzler shear force in five taurine cattle breeds. Anim. Genet. 43, 662–673. 10.1111/j.1365-2052.2012.02323.x 22497286 PMC3506923

[B60] Mészáros G. Fornara M. Reyer H. Wimmers K Solkner J. Brem G. (2019). Elevated haplotypes frequencies reveal similarities for selection signatures in Western and Russian Simmental populations. J. Cent. Eur. Agric. 20, 1–1. 10.5513/JCEA01/20.1.2412

[B61] Muniz M. M. M. Caetano A. R. Mcmanus C. Cavalcanti L. C. G. Façanha D. A. E. Leite J. H. G. M. (2016). Application of genomic data to assist a community-based breeding program: a preliminary study of coat color genetics in Morada Nova sheep. Livest. Sci. 190, 89–93. 10.1016/j.livsci.2016.06.006

[B62] Nakanishi T. Tokunaga T. Ishida T. Kobayashi I. Katahama Y. Yano A.. (2019). Changes in expression of the autophagy-related genes microtubule-associated protein 1 light chain 3β and autophagy related 7 in skeletal muscle of fattening Japanese Black cattle: a pilot study. Asian-Austral. J. Anim. Sci. 32:592. 10.5713/ajas.18.0370 30208695 PMC6409458

[B63] Nayeri S. Sargolzaei M. Abo-Ismail M. K. (2016). Genome-wide association for milk production and female fertility traits in Canadian dairy Holstein cattle. BMC Genet. 17:75. 10.1186/s12863-016-0386-1 27287773 PMC4901445

[B64] Nayeri S. Schenkel F. Fleming A. Kroezen V. Sargolzaei M. Baes C.. (2019). Genome-wide association analysis for β-hydroxybutyrate concentration in Milk in Holstein dairy cattle. BMC Genet. 20:58. 10.1186/s12863-019-0761-9 31311492 PMC6636026

[B65] Nayeri S. Stothard P. (2016). Tissues, metabolic pathways and genes of key importance in lactating dairy cattle. Springer Sci. Rev. 4, 49–77. 10.1007/s40362-016-0040-3

[B66] O' Connor S. F. Tatum J. D. Wulf D. M. Green R. D. Smith G. C. (1997). Genetic effects on beef tenderness in *Bos indicus* composite and *Bos taurus* cattle. J. Anim. Sci. 75, 1822–1830. 10.2527/1997.7571822x 9222838

[B67] Oleksyk T. K. Smith M. W. O'Brien S. J. (2010). Genome-wide scans for footprints of natural selection. Philos. Trans. R Soc. London B Biol. Sci. 365, 185–205. 10.1098/rstb.2009.0219 20008396 PMC2842710

[B68] Paim T. D. P. Hay E. H. A. Wilson C. Thomas M. G. Kuehn L. A. Paiva S. R.. (2020). Dynamics of genomic architecture during composite breed development in cattle. Anim. Genet. 51, 224–234. 10.1111/age.12907 31961956 PMC7065137

[B69] Patterson N. Price A. L. Reich D. (2006). Population structure and eigenanalysis. PLoS Genet. 2:e190. 10.1371/journal.pgen.0020190 17194218 PMC1713260

[B70] Pico B. A. (2004). Estimation of genetic parameters for growth traits in South African Brahman cattle (Magister Thesis). University of the Free State, South Africa. 10.4314/sajas.v34i6.3827

[B71] Pienaar L. Grobler J. P. Scholtz M. M. Swart H. Ehlers K. Marx M.. (2018). Genetic diversity of the Afrikaner cattle breed. Trop. Anim. Health Pro. 50, 399–404. 10.1007/s11250-017-1447-9 29043474

[B72] Purcell S. Neale B. Todd-Brown K. Thomas L. Ferreira M. A. Bender D.. (2007). PLINK: a tool set for whole-genome association and population-based linkage analyses. Am. J. Hum. Genet. 81, 559–575. 10.1086/519795 17701901 PMC1950838

[B73] Qu K. X. Zhu F. X. Wu G. S. Nie L. Jin X. D. Yang G. R.. (2006). Genetic diversity and population structure of BMY and brahman cattle revealed by six microsatellite loci. Hereditas 28, 285–290. 16551594

[B74] Raj A. Stephens M. Pritchard J. K. (2014). FastSTRUCTURE: variational inference of population structure in large SNP data sets. Genet. 197, 573–589. 10.1534/genetics.114.164350 24700103 PMC4063916

[B75] Raven L. A. Cocks B. G. Hayes B. J. (2014). Multibreed genome wide association can improve precision of mapping causative variants underlying milk production in dairy cattle. BMC Genomics 15:62. 10.1186/1471-2164-15-62 24456127 PMC3905911

[B76] Reed D. H. Frankham R. (2003). Correlation between fitness and genetic diversity. Conserv. Biol. 17, 230–237. 10.1046/j.1523-1739.2003.01236.x

[B77] Ríos-Utrera Á. Villagómez-Amezcua Manjarrez E. Zárate-Martínez J. P. Calderón-Robles R. C. Vega-Murillo V. E. (2020). Reproductive analysis of Brown Swiss x Zebu and Simmental x Zebu cows in tropical conditions. Rev. MVZ Córdoba 25:1637 10.21897/rmvz.1637

[B78] Sadri H. Giallongo F. Hristov A. N. Werner J. Lang C. H. Parys C.. (2016). Effects of slow-release urea and rumen-protected methionine and histidine on mammalian target of rapamycin (mTOR) signaling and ubiquitin proteasome-related gene expression in skeletal muscle of dairy cows. J. Dairy Sci. 99, 6702–6713. 10.3168/jds.2015-10673 27179859

[B79] Sanarana Y. Visser C. Bosman L. Nephawe K. Maiwashe A. van Marle-Köster E. (2016). Genetic diversity in South African Nguni cattle ecotypes based on microsatellite markers. Trop. Anim. Health Pro. 48, 379–385. 10.1007/s11250-015-0962-9 26611262

[B80] Schatz T. J. Thomas S. Geesink G. (2014). Comparison of the growth and meat tenderness of Brahman and F1 Senepol× Brahman steers. Anim. Prod. Sci. 54, 1867–1870. 10.1071/AN14243

[B81] Scheet P. Stephens M. (2006). A fast and flexible statistical model for large-scale population genotype data: applications to inferring missing genotypes and haplotypic phase. Am. J. Hum. Genet. 78, 629–644. 10.1086/502802 16532393 PMC1424677

[B82] Scholtz M. M. Bester J. Mamabolo J. M. Ramsay K. A. (2008). Results of the national cattle survey undertaken in South Africa, with emphasis on beef. Appl. Anim. Husb. Rural Dev. 1, 1–9.

[B83] Sharma A. Park J. E. Chai H. H. Jang G. W. Lee S. H. Lim D. (2017). Next generation sequencing in livestock species-a review. J Anim. Breed. Genomics 1, 23–30. 10.12972/jabng.20170003

[B84] Smith A. M. J. (2010). Genetic Analyses of Growth Traits for the Simbra Composite Breed (Doctoral dissertation, Stellenbosch: University of Stellenbosch).

[B85] Sölkner J. Filipcic L. Hampshire N. (1998). Genetic variability of populations and similarity of subpopulations in Austrian cattle breeds determined by analysis of pedigrees. Anim. Sci. 67, 249–256. 10.1017/S1357729800010006

[B86] Sulem P. Gudbjartsson D. F. Stacey S. N. Helgason A. Rafnar T. Magnusson K. P.. (2007). Genetic determinants of hair, eye and skin pigmentation in Europeans. Nat. Genet. 39, 1443–1452. 10.1038/ng.2007.13 17952075

[B87] Taberlet P. Valentini A. Rezaei H. R. Naderi S. Pompanon F. Negrini R.. (2008). Are cattle, sheep, and goats endangered species? Mol. Ecol. 17, 275–284. 10.1111/j.1365-294X.2007.03475.x 17927711

[B88] Tang K. Thornton K. R. Stoneking M. (2007). A new approach for using genome scans to detect recent positive selection in the human genome. PLoS Biol. 5:e171. 10.1371/journal.pbio.0050171 17579516 PMC1892573

[B89] Taye M. Kim J. Yoon S. H. Lee W. Hanotte O. Dessie T.. (2017). Whole genome scan reveals the genetic signature of African Ankole cattle breed and potential for higher quality beef. BMC Genet. 18:11. 10.1186/s12863-016-0467-1 28183280 PMC5301378

[B90] Tizioto P. C. Decker J. E. Taylor J. F. Schnabel R. D. Mudadu M. A. Silva F. L.. (2013). Genome scan for meat quality traits in Nelore beef cattle. Physiol. Genomics 45, 1012–1020. 10.1152/physiolgenomics.00066.2013 24022219

[B91] Utrera Á. R. Murillo V. E. V. Bermúdez M. M. Velázquez G. M. Ponce S. I. R. (2018). Genetic diversity assessment of the Mexican Simmental population through pedigree analysis. Rev. Bras. Zootecn. 47:88 10.1590/rbz4720160088

[B92] van der Westhuizen L. MacNeil M. D. Scholtz M. M. Neser F. W. Makgahlela M. L. van Wyk J. B. (2019). Genetic variability and relationships in nine South African cattle breeds using microsatellite markers. Trop. Anim. Health Pro. 6, 1–8. 10.1007/s11250-019-02003-z 31388877

[B93] Vitti J. J. Grossman S. R. Sabeti P. C. (2013). Detecting natural selection in genomic data. Annu. Rev. Genet. 47, 97–120. 10.1146/annurev-genet-111212-133526 24274750

[B94] Wathes D. C. Cheng Z. Chowdhury W. Fenwick M. A. Fitzpatrick R. Morris D. G.. (2009). Negative energy balance alters global gene expression and immune responses in the uterus of postpartum dairy cows. Physiol. Genomics 39, 1–13. 10.1152/physiolgenomics.00064.2009 19567787 PMC2747344

[B95] Wu X. Lund M. S. Sahana G. Guldbrandtsen B. Sun D. Zhang Q.. (2015). Association analysis for udder health based on SNP-panel and sequence data in Danish Holsteins. Genet. Sel. Evol. 47:50. 10.1186/s12711-015-0129-1 26087655 PMC4472403

[B96] Xia J. Qi X. Wu Y. Zhu B. Xu L. Zhang L.. (2016). Genome-wide association study identifies loci and candidate genes for meat quality traits in Simmental beef cattle. Mamm. Genome 27, 246–255. 10.1007/s00335-016-9635-x 27126640

[B97] Xu L. Bickhart D. M. Cole J. B. Schroeder S. G. Song J. Tassell C. P. V.. (2015). Genomic signatures reveal new evidences for selection of important traits in domestic cattle. Mol. Biol. Evol. 32, 711–725. 10.1093/molbev/msu333 25431480 PMC4441790

[B98] Yin H. Gui Y. Du G. Frohman M. A. Zheng X.-L. (2010). Dependence of phospholipase D1 multi-monoubiquitination on its enzymatic activity and palmitoylation. J. Biol. Chem. 285, 13580–13588. 10.1074/jbc.M109.046359 20189990 PMC2859519

[B99] Zhang Q. Calus M. P. Guldbrandtsen B. Lund M. S. Sahana G. (2015). Estimation of inbreeding using pedigree, 50k SNP chip genotypes and full sequence data in three cattle breeds. BMC Genet. 16:88. 10.1186/s12863-015-0227-7 26195126 PMC4509611

[B100] Zhang Z. Kargo M. Liu A. Thomasen J. R. Pan Y. Su G. (2019). Genotype-by-environment interaction of fertility traits in Danish Holstein cattle using a single-step genomic reaction norm model. Heredity (Edinb) 123, 202–214. 10.1038/s41437-019-0192-4 30760882 PMC6781120

